# Pronounced polarization-induced energy level shifts at boundaries of organic semiconductor nanostructures

**DOI:** 10.1038/ncomms9312

**Published:** 2015-10-06

**Authors:** K. A. Cochrane, A. Schiffrin, T. S. Roussy, M. Capsoni, S. A. Burke

**Affiliations:** 1 Department of Chemistry, University of British Columbia, Vancouver British Columbia, Canada V6T 1Z1; 2 Department of Physics and Astronomy, University of British Columbia, Vancouver British Columbia, Canada V6T 1Z1; 3 Quantum Matter Institute, University of British Columbia, Vancouver British Columbia, Canada V6T 1Z4

## Abstract

Organic semiconductor devices rely on the movement of charge at and near interfaces, making an understanding of energy level alignment at these boundaries an essential element of optimizing materials for electronic and optoelectronic applications. Here we employ low temperature scanning tunneling microscopy and spectroscopy to investigate a model system: two-dimensional nanostructures of the prototypical organic semiconductor, PTCDA (3,4,9,10-perylenetetracarboxylic dianhydride) adsorbed on NaCl (2 ML)/Ag(111). Pixel-by-pixel scanning tunneling spectroscopy allows mapping of occupied and unoccupied electronic states across these nanoislands with sub-molecular spatial resolution, revealing strong electronic differences between molecules at the edges and those in the centre, with energy level shifts of up to 400 meV. We attribute this to the change in electrostatic environment at the boundaries of clusters, namely via polarization of neighbouring molecules. The observation of these strong shifts illustrates a crucial issue: interfacial energy level alignment can differ substantially from the bulk electronic structure in organic materials.

## Abstract (web summary)

Interfaces are essential in organic semiconductor devices, yet the detailed connection between interface geometry and energy level alignment are not fully understood. Here, Cochrane *et al*. quantify energy level shifts with sub-molecular resolution within a nanoscale model organic semiconductor system.

## Graphical abstract

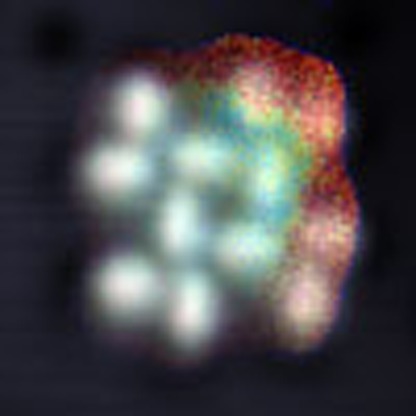

The electronic properties of interfaces often differ from the bulk; a characteristic that underlies many device applications. Organic materials are no different in this regard, where interfacial regions are narrowed to few-molecule length scales due to low dielectric constants^1^. Organic semiconductors are of great interest as they offer low-cost, light-weight and low embodied energy options for a broad range of electronic and optoelectronic applications, including photovoltaics^2^, light emitting diodes^3^ and field effect transistors^4^. These applications require understanding and control over interface electronic states^5^. For example, transport in pentacene thin-film transistors occurs within a few molecular layers of the gate^6^. The importance of interfaces for organic photovoltaic cells is even more striking, as free carrier generation relies on an offset of electronic states at the heterojunction interface to drive the separation of bound excitons^7^,^8^. Meanwhile, bulk measurements of organic semiconductors have indicated that distinct interfacial states are present in mixed systems^9^,^10^ as well as between different phases in a single component material^11^. Effects of the local electrostatic environment have also been shown to significantly shift molecular energy levels through polarization of adjacent matter^12^,^13^,^14^,^15^,^16^,^17^. Such effects can shift molecular states up to 500 meV^13^,^15^,^18^ and can contribute of order 1 eV to the transport gap of organic semiconductors^19^,^20^ with consequences for both transport and charge transfer across interfaces^21^. The array of effects involved, combined with often disordered interfaces, creates substantial challenges in designing optimized devices.

Elucidating the exact structure and electronic properties at these boundaries requires nanometer scale probes that are difficult to apply to a full device structure. Recent studies using scanning tunnelling microscopy (STM) and spectroscopy (STS), alongside complementary techniques, on mixed donor–acceptor monolayers on metallic and semi-metallic surfaces have sought to characterize their electronic structure as model interfaces for organic photovoltaic materials^22^,^23^,^24^,^25^. In these examples, energy level shifts in the electronic states of both species arise from a combination of intermolecular and molecule-substrate interactions. Although these well-ordered systems come close to mimicking photovoltaic device materials in a way that is amenable to surface science probes, the single-molecule ‘width' of the interfaces present in these layers, and the influence of a metallic substrate, creates an environment that deviates from actual devices and influences the measured electronic structure^26^,^27^.

Here we examine the boundaries of two-dimensional (2D) clusters of PTCDA (3,4,9,10-perylenetetracarboxylic dianhydride) to study nanoscale lateral interfaces (with the vacuum) involving just one component. PTCDA has been widely used as a prototypical organic semiconductor, due to its optoelectronic properties^28^,^29^ and tendency to order on a wide range of surfaces^30^,^31^,^32^,^33^,^34^. Studies of the electronic structure of PTCDA monolayers and thin films on metal substrates have shown that molecular energy levels can shift by 100s of meV due to a variety of effects including small differences in the hydrogen bond (H-bond) lengths between inequivalent adsorption sites^16^,^35^,^36^,^37^,^38^,^39^, and stabilization of charge by polarization of the surroundings as a function of film thickness and distance from an interface^13^. Using low-temperature STM and STS we have locally probed the structure and electronic states of PTCDA clusters that are decoupled from the Ag(111) substrate by a bilayer film of NaCl to allow examination of the intrinsic electronic effects within clusters on the molecular scale^40^. Pixel-by-pixel STS allows us to probe the energy levels of electronic states with sub-molecular spatial resolution, revealing the influence of the abrupt change in the local chemical and electrostatic environment at the edges of nanoislands.

## Results

### Topography and tunneling spectroscopy of isolated PTCDA molecules

**Figure 1:**
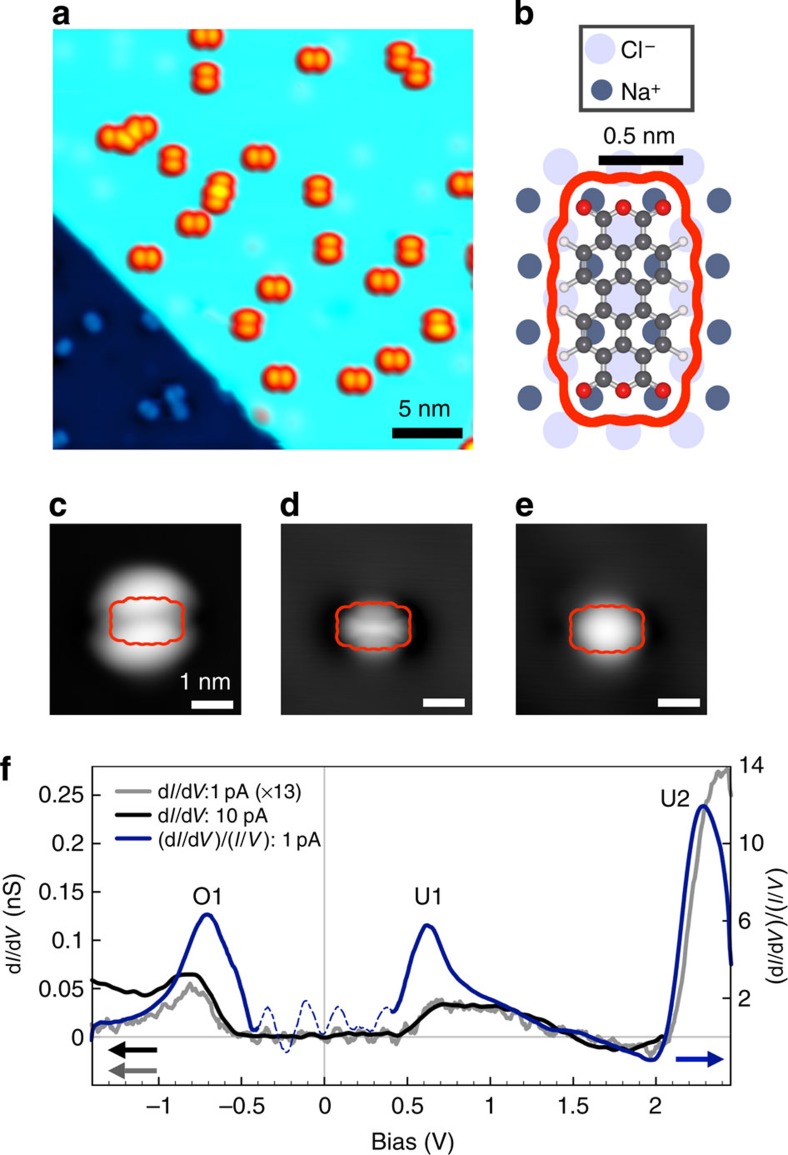
STM/STS of isolated PTCDA molecules on NaCl(2 ML)/Ag(111). (**a**) STM constant-current image (30 × 30 nm^2^, *I*=30 pA, *V*_*b*_=−1.5 V) of PTCDA adsorbed on NaCl(2 ML)/ Ag(111). (**b**) Adsorption geometry of PTCDA on the NaCl lattice. Electronic density isocontour (0.0004, eÅ^−3^) is outlined in orange (calculated in Gaussian using B3LYP unrestricted 6-31G). (**c**–**e**) STM images of an isolated PTCDA molecule at different bias voltages on NaCl (2 ML)/Ag(111) showing submolecular resolution (4 × 4 nm^2^, (**c**) *I*=50 pA, *V*_*b*_=−1.5 V, (**d**) *I*=40 pA, *V*_*b*_=+0.5 V and (**e**) *I*=40 pA, *V*_*b*_=+1.0 V). (**f**) STS spectra of an isolated PTCDA molecule on NaCl (2 ML)/Ag(111) (*I*=1 pA and 10 pA at *V*_*b*_=−1.5 V). The differential conductance (d*I*/d*V*) (rescaled by a factor of 13 for *I*=1 pA in grey) and normalized differential conductance (d*I*/d*V*)/(*I*/*V*) are shown (region near zero with divergences with dashed line). The Fermi energy *E*_F_ corresponds to *V*_*B*_=0 V. Three electronic resonances are identified as O1, U1 and U2. Images and STS at 4.3 K.

Figure 1a shows an STM topography of isolated PTCDA molecules deposited and imaged at ∼4.3 K on NaCl (2 ML) /Ag(111). On the NaCl terrace (upper right of Fig. 1a), two orthogonal orientations were observed, 45° from the NaCl(100) edge, corresponding to adsorption on the Cl^−^ top site previously identified for bulk NaCl^41^,^42^ (Fig. 1b, Supplementary Note 1). Isolated PTCDA molecules on Ag(111) were also seen. STM imaging at negative sample bias (occupied states) showed a double-lobed structure with a single nodal plane along the long symmetry axis (Fig. 1a,c), similar to that observed previously^43^,^44^,^45^. At positive biases (unoccupied states) PTCDA was imaged as a rounded oblong structure, with some substructure appearing at lower biases (Fig. 1d,e).

STS of isolated PTCDA molecules (Fig. 1f) showed three main features within the experimental voltage range (−1.5 V to +2.8 V). We observed a well-defined tunnelling resonance at −0.7 V (O1), a broad feature at ∼+0.6 V (U1), and a strong resonance with an onset at ∼2 V (U2). Peak energies were found to be independent of the tip-molecule distance, indicating there was no observable effect of the electric field in the tunnel junction (Fig. 1f, black and grey)^46^. The negative differential conductance above the U1 state is consistent with an increasing bias-dependent barrier height^46^. The normalized differential conductance ((d*I*/d*V*)/(*I*/*V*)) is used for the remaining discussion, as it reduces the exponential background for the bias range considered, provides a closer approximation to the local density of states, and facilitates peak identification (see Methods for details)^47^,^48^.

From comparison with previous work^45^, we expect that PTCDA on NaCl(2 ML)/Ag(111) is negatively charged (Supplementary Note 2). This results in the partial occupation of the lowest occupied molecular orbital (LUMO). Unperturbed, this half occupied state should appear at the Fermi energy (*E*_*f*_). However, the addition of a charged particle (electron addition or removal) to this system, such as in measurements by electron spectroscopies (STS, photoemission), results in splitting of the state due to Coulomb repulsion into peaks above and below *E*_*f*_, separated by the Hubbard energy, U. Values of U in PTCDA have been determined previously, and range from 3 to 0.25 eV depending on whether the molecule is in the gas phase, or adsorbed on a surface where electrons can screen charge^49^,^50^. Here we tentatively identify the first unoccupied resonance above *E*_*f*_, U1, as the upper Hubbard state. We believe the lower Hubbard state is close in energy to or below the HOMO, overlapping to form the resonance O1 as no state observed was spatially similar to U1 (ref. 51). This places the screened Hubbard U at ≳1.2 eV for the isolated molecule on a NaCl bilayer on Ag(111). Resonance U2 was tentatively identified as a combination of the nearly degenerate LUMO+1 and LUMO+2 (ref. 37). However, we note that recently Liljeroth at al. found that the resonances in tunnelling spectra of cobalt phthalocyanine on an insulator could not be identified by a single-particle molecular orbital calculation. When many-body effects were taken into consideration the identified states were not only shifted in energy but also reordered^52^ adding further complexity to the task of assigning electronic resonances to specific molecular orbitals in tunnelling spectra. As such, definitive assignment of the states is beyond the scope of this work, and we therefore refer to these states in the following as the empirically labeled O1, U1 and U2.

### Structure of PTCDA nanoislands

**Figure 2:**
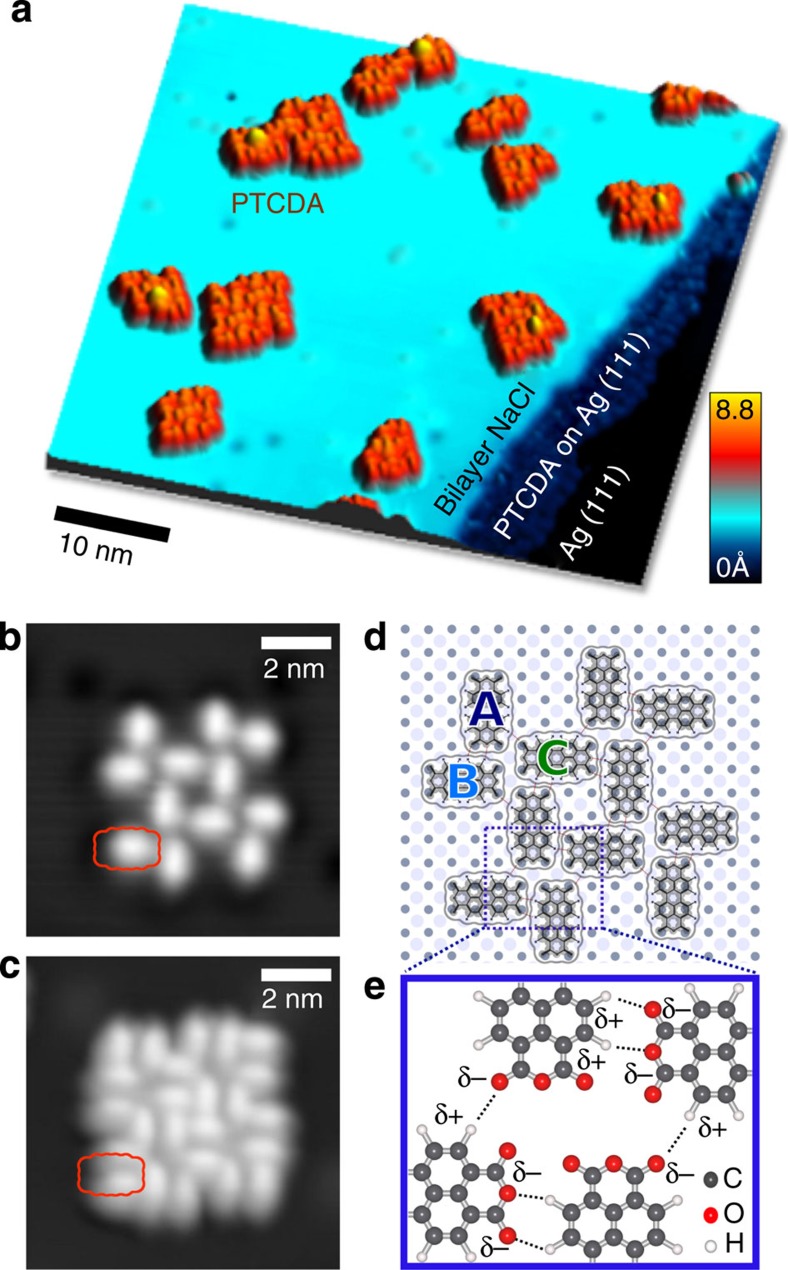
STM imaging and structure of PTCDA 2D clusters on NaCl(2 ML)/Ag(111). (**a**) STM constant-current image (45 × 45 nm^2^, *I*=30 pA, *V*_*b*_=−1.5 V) of PTCDA clusters on NaCl(2 ML)/Ag(111) formed after room temperature annealing. (**b**,**c**) STM images of a 12-molecule PTCDA nanoisland (9 × 9 nm^2^, *I*=30 pA, (**b**) *V*_*b*_=+1.0 V and (**c**) *V*_*b*_=−1.5 V). Images at 4.3 K. (**d**) Structural model of 12-molecule island showing positions on NaCl lattice. (**e**) Model of the hydrogen bond network within the nanoisland. Partial charges (*δ*+, *δ*−) are shown.

When annealed at room temperature, the isolated molecules in Fig. 1a diffuse to form 2D clusters, as shown in Fig. 2a. These nanoislands, exhibiting different sizes and structural arrangements, are well ordered and have defined edges that serve as a model for an abrupt interface. In Fig. 2b–d, we consider a 12-molecule island. On the basis of the observed registry with respect to the NaCl lattice and the orthogonal orientation of molecules within the cluster, we deduce that the NaCl adsorption configuration is the same as for isolated PTCDA (compare Fig. 1b and Fig. 2d).

Molecules can be classified into three categories (A, B, C), based on their position (edge or centre) within the 12-molecule nanoisland and the degree of interaction with surrounding molecules. Edge molecules of type A and B differ in the number of nearest neighbours: type A with two, type B with three. Both A and B each have three H-bonds with two neighbouring PTCDA molecules. Type A has one H-donor (proton donating) and two H-acceptors (proton accepting). Type B has two H-donors and one H-acceptor. Type B is also positioned head-to-head with another PTCDA molecule where the electron rich anhydride groups face each other. Centre molecules, C, are fully surrounded by five PTCDA molecules and form six individual H-bonds, with three H-donors and three H-acceptors. They also each have a single head-to-head anhydride interaction.

### Tunneling spectroscopy of PTCDA nanoislands

**Figure 3:**
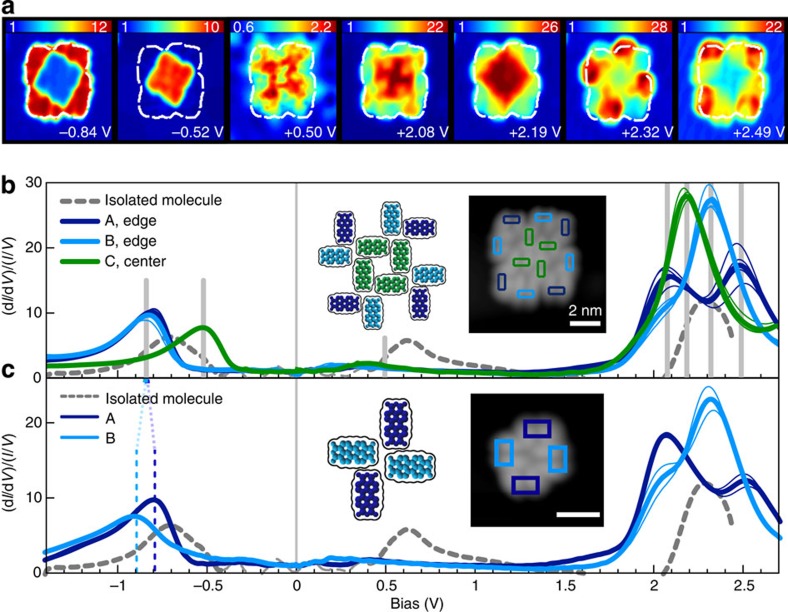
STS of PTCDA nanoislands. (**a**) STS maps of a 12-molecule PTCDA island at increasing sample bias (8.5 × 8.5 nm^2^, *V*_*b*_=−0.84, −0.52, +0.50, +2.08, +2.19, +2.49 V). Colour scales represent (d*I*/d*V*)/(*I*/*V*), dotted white line indicates outline of molecular cluster. (**b**) (d*I*/d*V*)/(*I*/*V*) spectra for molecule types A, B, C within the 12-molecule cluster (blue, cyan and green). Thick curves represent an average over all equivalent molecules. Thin curves are averaged over individual molecules. Grey vertical lines denote bias voltages of the STS maps in **a**. Inset: STM topographic scan taken during STS grid acquisition with spatially averaged spectra locations represented by coloured boxes (8.5 × 8.5 nm^2^, *I*=30 pA, *V*_*b*_=−1.5 V). (**c**) (d*I*/d*V*)/(*I*/*V*) spectra of a four-molecule island. Inset: STM topographic scan (6 × 6 nm^2^, *I*=30 pA, *V*_*b*_=−1.5 V).

To examine the local electronic structure of PTCDA clusters, we performed STS as a function of position (*x*,*y*) on the sample to construct a 3D grid of [(d*I*/d*V*)/(*I*/*V*)](*x*,*y*,*V*) (see Fig. 3 and Methods). The electronic features of the isolated molecule (Fig. 1f) differs from those of PTCDA in a nanoisland (regardless of type A, B or C). U1 is significantly reduced in intensity and possibly broadened (Fig. 3b) for all positions and appears spatially delocalized in the corresponding STS map at *V*_B_=0.50 V. The maximum intensity of U1 shifts towards *E*_*f*_, consistent with a reduction of the Hubbard U due to screening by neighbouring molecules^53^. Each of the structurally equivalent positions are also electronically equivalent, with all four molecules of the same type (A, B, or C) showing nearly identical (d*I*/d*V*)/(*I*/*V*) signatures (Fig. 3b, thin curves). However, the relative position within the cluster does influence the spectroscopic signature for each type of site. For the occupied state O1, a clear difference is seen between centre and edge molecules (see Fig. 3b): A and B show a shift away from *E*_*f*_ with respect to a single PTCDA molecule (dashed line), while the centre molecule C shows a shift towards *E*_*f*_. These shifts produce a ∼300 meV difference in the energy of O1 between edge and centre molecules. The spatially resolved STS maps corresponding to the peak voltages of O1 for the edge and centre molecules, Fig. 3a *V*_*b*_=−0.84 V and *V*_*b*_=−0.53 V, respectively, show the clear spatial separation of the local density of states at these energies. The unoccupied resonance U2 shows a similar onset for all three molecular positions (∼2 V). However, the peak intensities and positions differ for A, B and C. The corresponding STS maps (Fig. 3a for *V*_*b*_=2.08 V, 2.19 V, 2.32 V and 2.49 V and Supplementary Movie for full bias range of −1.5 to 2.7 V) clearly show this position dependence.

It is important to note that molecules of the same type (that is, with equivalent molecular environments) do not have equivalent adsorption sites with respect to the Moiré pattern resulting from the lattice mismatch between NaCl (2 ML) and the underlying Ag(111) lattice^54^ (see Supplementary Fig. 1 and Note 3). These spatial variations in substrate electronic structure and electrostatic environment have a negligible influence, indicating that intermolecular interactions dominate in generating the observed shifts in molecular electronic states.

For comparison, a four-molecule island (Fig. 3c, Supplementary Fig. 2) with the same type A and B H-bonding motif seen in the larger cluster was found. Types A and B differ between the two islands only in next-nearest neighbour, where these are absent in the four-molecule island. Feature O1 for A and B in the four-molecule island differs by an energy of ∼100 meV. In contrast, O1 is indistinguishable for A and B in the 12-molecule cluster, within the measurement resolution (12 meV). The energy onset of U2 in A and B does not change from the 4- to 12-molecule island, although the relative peak intensities of the close-lying states above ∼2 eV are influenced by the presence or absence of a next-nearest neighbour. The influence of next-nearest neighbours on the local electronic structure provides evidence of longer ranged interactions, either via through-space electrostatic effects or via weak electronic hybridization within the planar PTCDA structure.

**Figure 4:**
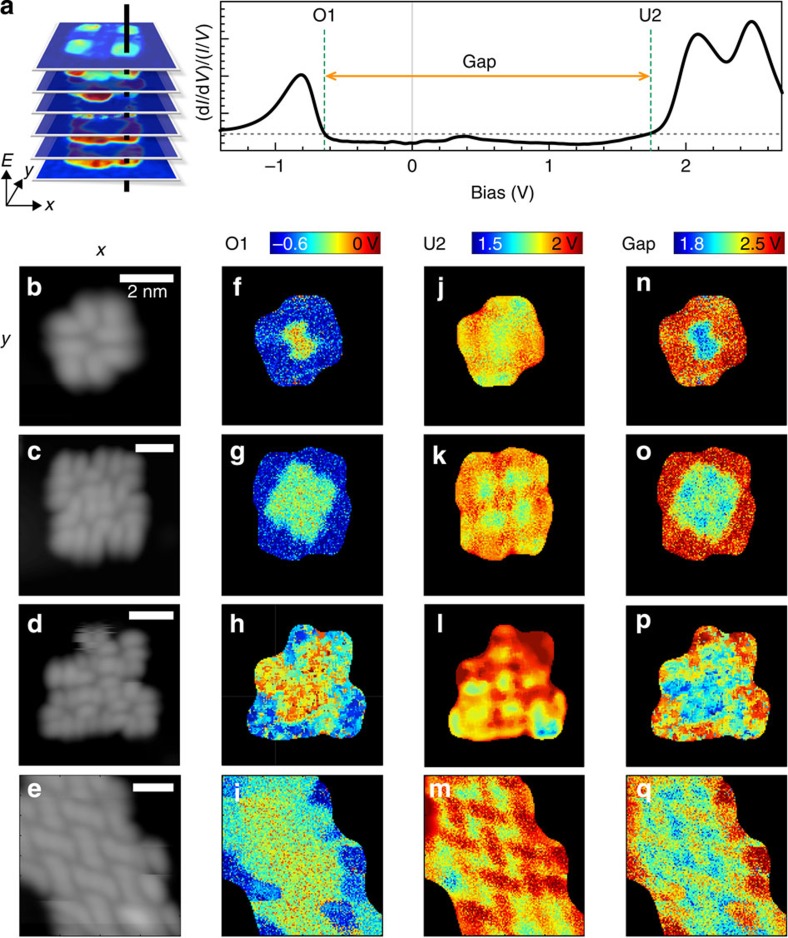
Local energy level alignment within PTCDA islands of different sizes. (**a**) For each (d*I*/d*V*)/(*I*/*V*) spectrum acquired at a given tip position (*x*,*y*), ‘band edges' are defined as the voltage onsets ((d*I*/d*V*)/((*I*/*V*)=3)) of states O1 and U2. The band gap is the voltage difference between these onsets. (**b**–**e**) STM topographic images acquired during spectroscopic measurement for a 4-molecule, 12-molecule, 18-molecule island and herringbone nanoribbon, respectively (*I*=30 pA*, V*_*b*_=−1.5 V, (**b**) 6 × 6 nm^2^, (**c**) 8.5 × 8.5 nm^2^, (**d**) 9.5 × 9.5 nm^2^, (**e**) 8 × 8 nm^2^). (**f**–**i**) 2D (*x*,*y*)-dependent maps of O1 voltage onset for (**f**) 4-molecule island, (**g**) 12-molecule island, (**h**) 18-molecule island and (**i**) herringbone nanoribbon. (**j**–**m**) Corresponding 2D (*x*,*y*)-dependent maps of voltage onset of U2. (**n**–**q**) Corresponding 2D (*x*,*y*)-dependent maps of band gaps.

To visualize the resulting energy level alignment within PTCDA clusters, the voltage onsets of O1 and U2 were determined for each pixel in four different islands, Fig. 4b–e. We define the corresponding ‘band gap' as the voltage difference between these two onsets (Fig. 4a). Electronic features O1 and U2 were chosen due to their correspondence with the transport gap measured by photoemission^13^ and the strong suppression of the mid-gap U1 state in clusters. The corresponding gap maps (Fig. 4n–q) show a difference of ∼400 meV between the centre and edge molecules. This is dominated by a downward shift of O1 for the edge molecules relative to the centre molecules, influencing both the gap and energy level alignment at this interface. Notably, different PTCDA adsorption motifs (for example, herringbone in Fig. 4e, open square phase b,c) show similar energy shifts of the electronic features at the edges.

### Microelectrostatic calculations of polarization energy

To investigate the influence of the local, inhomogeneous electrostatic environment, we consider the site-specific stabilization of the charge added or removed during tunneling spectroscopy by the nearly instantaneous electronic polarization of all other molecules in the cluster^53^. This stabilization of the transient charge in a single-particle spectroscopy, such as STS or photoemission, results in a decrease in the measured ionization potential and increase in the electron affinity, narrowing the transport gap observed. A microelectrostatic model was used to calculate the stabilization energy of a single point charge placed at each molecular position due to the electronic polarization of the cluster (Supplementary Method). The in-plane, anisotropic polarizabilty of PTCDA, *α*_MM_=50.3 Å^3^ (short axis) and *α*_LL_=88.2 Å^3^ (long axis), previously calculated by density functional theory was used^55^. Note that this model does not take into account the influence of the underlying substrate, and neglects the spatial distribution of the charge within the molecule when a given orbital is probed. The polarization energy, *E*_p_, for each PTCDA site due to the anisotropic response of neighbouring molecules within the island can be expressed as:


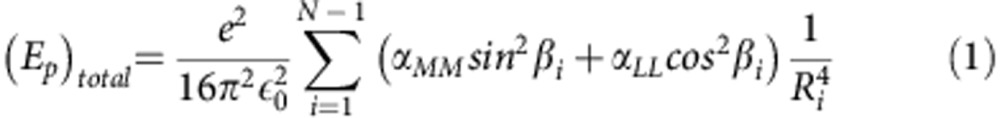


where *R* is the distance between the pair considered, and *β* is the angle between the vector connecting the pair and the long axis of the molecule. The total polarization energy for each site due to the response of a cluster of size *N* is calculated by summing *E*_*p*_ for all other molecules (Supplementary Fig. 3 and Methods for further detail).

**Figure 5:**
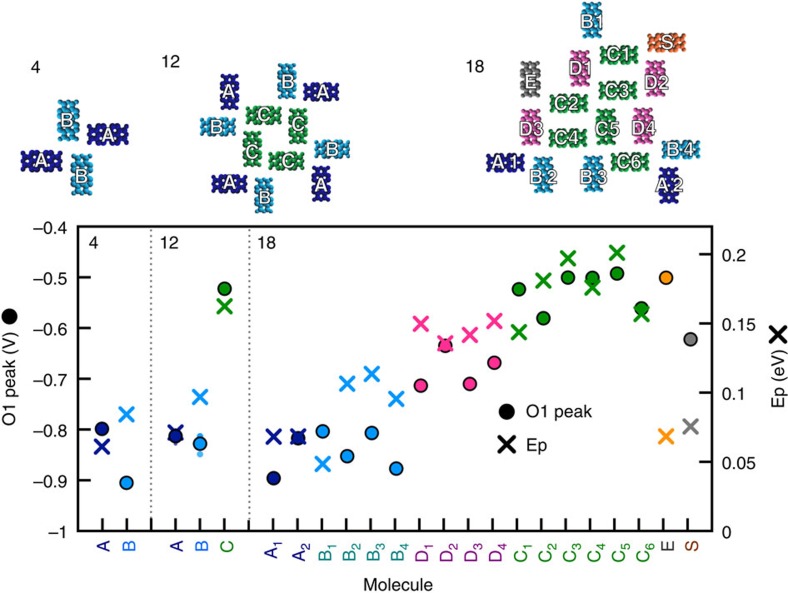
Occupied state of PTCDA islands with polarization energy. The result of the microelectrostatic calculation (crosses) was plotted for each molecule in three islands: 4-molecule, 12-molecule and 18-molecule, and compared with the peak of the O1 state (dots). The specific molecules within the islands identified on the *x* axis are labelled in the schematic structure of each cluster above. The vertical axes for O1 and *E*_p_ differ by a factor of 2.8.

The results of this calculation for three different cluster sizes and geometries (those in Fig. 4b–d) are shown in Fig. 5 and compared with the measured positions of the O1 state. The qualitative trends observed in the data for different types of sites in each of the clusters are generally well reproduced by the shifts predicted by the polarization energy calculation across all three cluster sizes and geometries. For example, for the 12-molecule island (shown in Fig. 3a,b, and Fig. 4c,g,k,o), the centre (C) molecules show an O1 state that is closer to *E*_*f*_ than the A/B edge molecules, corresponding to the larger polarization energy calculated for these positions compared with the A/B edge sites. Even more strikingly, for the 18-molecule island (Fig. 4d,h,l,p and Supplementary Fig. 4 and Note 4), nearly all 18 sites, each unique in geometry, follow the overall trend predicted by the polarization calculation. Here A, B and C-type positions bear resemblance to the A, B, and C identified in the 4- and 12-molecule clusters in terms of nearest neighbours and H-bonding, while D-type molecules lie at an edge, but are more fully surrounded than either A or B-type sites. The O1 states measured for D-type positions were noted to lie between those of the centre molecules and A/B edge molecules, which is similarly reproduced in the calculation. Two outliers labelled S and E showed atypical spectroscopic behaviour that we attribute to defects in the cluster or underlying substrate. In these calculations, all molecules in the cluster were considered. We find that next-nearest neighbours each contribute a few percent to the total *E*_*P*_, while next-next-nearest neighbours still contribute ∼0.5% each to the total *E*_*P*_ (see Supplementary Fig. 4 for example). While the shifts in energy levels are strongly localized to the edge, the effect arises from the interaction with molecules several sites away.

**Figure 6:**
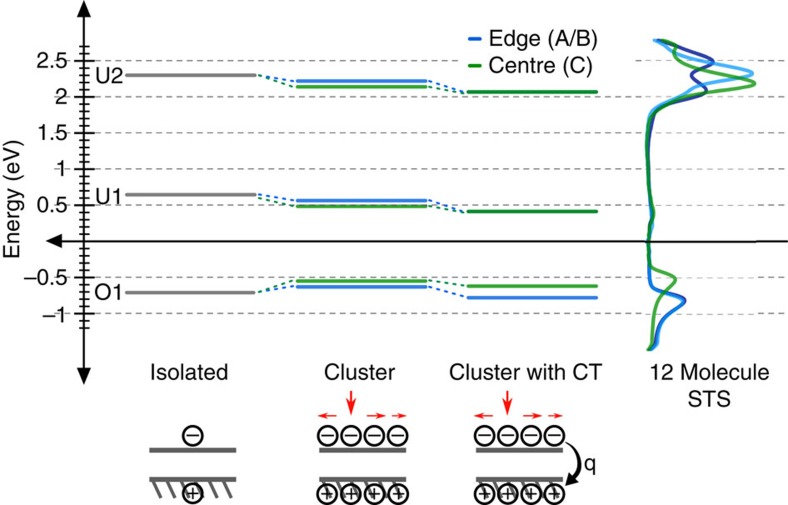
Comparison of calculated and measured edge and centre states of PTCDA clusters. Electronic state energies relative to *E*_*f*_ derived from the measured spectrum of the isolated molecule (left), applying the calculated polarization energies for the edge (blue) and centre (green) of the 12-molecule cluster (middle), and accounting for the observed charge transfer as a rigid shift of the energy levels and compared to the experimental STS (left). Red arrows above clusters depict the induced polarization after addition of a tunnelling electron.

Although the polarization energy need not be equal for both the removal and addition of charge^56^, we expect a shift of the unoccupied states due to this effect as well. The onset of U2 does shift towards *E*_*f*_ for all sites relative to the isolated molecule spectrum, however, there is minimal spatial variation of the U2 onset. The onset of the O1 state for A/B edge molecules also lies below the O1 onset for the single-molecule spectrum, implying an overall downward shift of the spectra indicative of a charge transfer between the cluster and the underlying Ag(111) substrate. As in mixed monolayers, this charge transfer may be spatially varying due to the different relative shifts of the U1 and U2 states for different sites with an alignment of the unoccupied states, here mediated by the U1 mid-gap state. Recent theoretical literature has demonstrated site-specific charge transfer on a molecule/insulator/metal system leading to spatially varying charge on molecules within a layer with an insulating barrier to the metal substrate^57^. Assuming equal polarization energies for electron addition and removal, and a site-specific charge transfer that aligns the U2 states as observed, there is a nearly quantitative agreement between the measured and predicted position of the O1 state (Fig. 6).

## Discussion

The remarkably good agreement between the experimental data and the predictions of the microelectrostatic model, for clusters of different size and geometry, provides compelling evidence that the mechanism dictating the observed site-specific energy levels arises from the stabilization of charges by the electronic polarization of neighbouring PTCDA molecules. The calculation neglects several other possible origins of energy level shifts including: conformational differences due to differing intermolecular interactions, in-plane hybridization, and differences in H-bonding. Notably, subtle differences in hydrogen bonding between adjacent PTCDA molecules have been implicated in ongoing discrepancies between experimental and DFT results for PTCDA monolayers on Ag(111) and Ag(100)^37^,^38^, highlighting some of the computational challenges inherent in interfacial systems, particularly where covalent and non-covalent interactions compete and electronic correlations cannot be neglected. These effects, and the inhomogeneous intramolecular charge distribution within the molecule, neglected in the calculation, may explain the deviations between the predicted and measured O1 position where *E*_p_ differences are small, such as the reversal of the order of states A and B. Nevertheless, this simple electronic polarization picture adequately captures the gross features while being computationally straightforward and tractable for large systems where *ab initio* formalisms including van der Waals interactions and correlations would be inaccessible. The ability to study large systems via an analytic model where electronic polarization plays a dominant role opens up the possibility of studying realistic interfacial systems, including the effect of disorder, by determining only the position, orientation and anisotropic polarizability of the molecular components.

The correspondence between the measured electronic energy level shifts and the calculated polarization energies is aided by the weak in-plane hybridization of PTCDA^29^, as one can adequately describe the injection and removal of charges by a localized molecular ion. Although Temirov *et al*.^58^ observed a delocalized interface state arising from the interaction of PTCDA with the metallic substrate, the NaCl bilayer used here suppresses molecule–substrate hybridization, and our observations are expected to be representative of electronic effects that can be attributed to the intrinsic molecular and intermolecular interactions within the PTCDA clusters. As the majority of organic semiconductors are characterized by weak orbital overlap (small hopping integrals) and conjugated molecules typically have large polarizabilities, these electronic polarization effects are expected to be significant, if not dominant, relative to other interfacial electronic effects in many cases.

Here local topographic and spectroscopic measurements performed with sub-molecular resolution on 2D nanoscale clusters of PTCDA have revealed a striking difference between the electronic states of molecules residing at the edges of these clusters and those in the centre. Edge molecules exhibit a gap that is up to 400 meV larger than observed for inner molecules (representative of a 2D ‘bulk'), arising primarily from a shift in the occupied state energies that correspondingly influences level alignment for a boundary region of single-molecule width. These site-specific energy level shifts measured by STS arise from differences in the local electronic polarization environment provided by the cluster that responds instantaneously to a transient, localized charge. Electronic polarization effects are expected to strongly influence hopping-like transport and processes such as photoinduced charge separation at heterojunction interfaces^59^ where a transient molecular ion is formed. As the polarizability of most organic semiconductors is anisotropic, both the local structure and orientation of molecules at interfaces will play a significant role in the resulting energy level alignment. Yet, where these effects dominate, as they do in planar arrangements of PTCDA, an accessible model can be used to predict interfacial energy level shifts. This model can easily be extended to large and more complex systems to address issues related to interface geometry and disorder, as well as identify potential optimization paths through careful design of interface interactions.

## Methods

### Sample preparation

The Ag(111) surface (Mateck GmbH) was prepared in ultrahigh vacuum (UHV) by repeated cycles of Ar^+^ sputtering and annealing at 770 K. NaCl (TraceSELECT ≥99.999%, Fluka), was evaporated at ∼800 K onto the sample held at 370 K, resulting in (001) bilayer islands covering ∼50% of the surface. PTCDA (98%, Alfa Aeasar) was thermally deposited at 550 K onto the NaCl/Ag(111) surface held between 4.2 and 4.5 K. Islands of 2 to ∼40 molecules were formed via subsequent annealing to room temperature.

### Scanning tunneling microscopy and spectroscpy

STM and STS measurements were performed in UHV at ∼4.3 K (Omicron Nanotechnology) with an Ag-terminated Pt/Ir (Goodfellow) tip, verified as metallic on the bare Ag(111) substrate with a flat DOS and the onset of the Ag (111) surface state at −65 mV. STM topographic images were acquired in constant-current mode. The bias voltage relative to the sample is reported throughout the text. (d*I*/d*V*)/(*I*/*V*) data as a function of sample bias voltage were obtained by numerically differentiating *I*(*V*) curves measured for each (*x*, *y*) tip position on the surface (128 by 128 pixels in *x*–*y*, 512 bias points taken for the voltage range scanned, resulting in ∼8 mV resolution) with the feedback loop disabled. *I*/*V* and (d*I*/d*V*)/(*I*/*V*) spectra were smoothed with a three-point moving average filter (Supplementary Fig. 5) giving an energy resolution of 12 meV. Normalized differential conductance ((d*I*/d*V*)/(*I*/*V*)) minimizes the exponential background in the tunnelling current *I*(*V*) given by the transmission function at energies away from the Fermi energy (*E*_f_) (refs 48, 49). The normalization does not introduce additional features in the spectra, except for points near *V*_b_=0 where the divergence was removed (Fig. 1f).

## 

## Additional information

**How to cite this article:** Cochrane, K. A. *et al*. Pronounced polarization-induced energy level shifts at boundaries of organic semiconductor nanostructures. *Nat. Commun.* 6:8312 doi: 10.1038/ncomms9312 (2015).

## Supplementary Material

Supplementary Information Supplementary Figures 1-5, Supplementary Notes 1-4, Supplementary Methods and Supplementary References.

Supplementary Movie 1 Evolution of states of a 12-molecule PTCDA island. Pixel-by-pixel scanning tunneling spectroscopy maps of a 12-molecule island at increasing sample bias (8.5 x 8.5 nm^2^, Vb = −1.5 to 2.7 V).
