# Supplementary material for: Pronounced polarization-induced energy level shifts at boundaries of organic semiconductor nanostructures
Source: Nat Commun. 2015 Oct 6;6:8312. doi: 10.1038/ncomms9312 (PMC4600718; doi:10.1038/ncomms9312)
Supplement: Supplementary Information — Supplementary Figures 1-5, Supplementary Notes 1-4, Supplementary Methods and Supplementary References. [file ncomms9312-s1.pdf]

Supplementary Figures

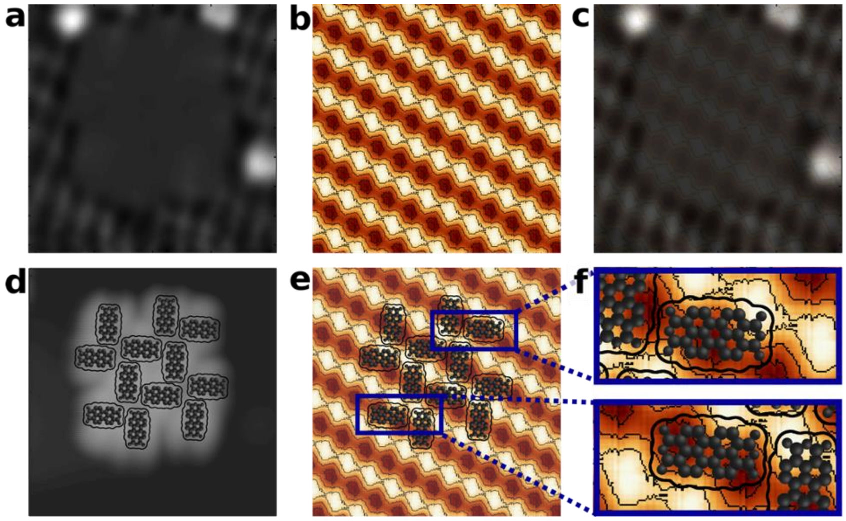

Supplementary Figure 1. Adsorption of 12-molecule island with respect to Moiré pattern of NaCl(2 ML)/Ag(111). a, (dI/dV)/(I/V) STS map of 12-molecule island at V_b=1.45V showing underlying Moiré pattern (8.5 x 8.5 nm^2, I_t = 30 pA). **b,** NaCl(2 ML)/Ag(111) Moiré pattern obtained by Fourier-filtering the image components in (a) corresponding to the PTCDA island. **c,** Overlay of (a) and (b). **d,** STM topography (V_b = -1.5 V, I_t = 30 pA) corresponding to STS map in (a). **e,** Adsorption positions of molecules with respect to Moiré. **f,** Spectroscopically equivalent PTCDA molecules of type A (see main text) showing inequivalent adsorption sites with respect to the Moiré. All constant-current STM images were processed with Wsxm.[5]

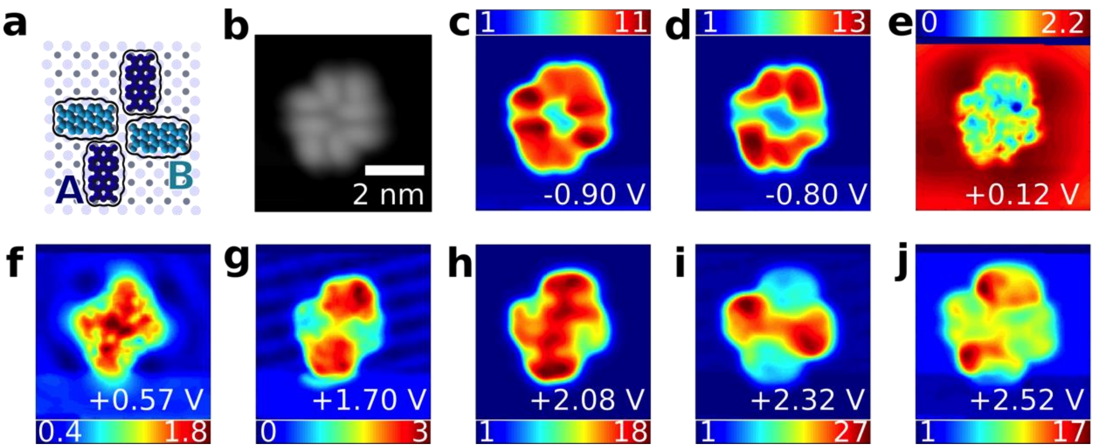

Supplementary Figure 2. 4-molecule nano-island on NaCl(2 ML)/Ag(111). a, Molecular arrangement on NaCl lattice. **b,** Constant current STM image (6 x 6 nm^2, V_b = -1.5 V, I_t = 30 pA). **c-j,** Corresponding (dI/dV)/(I/V) STS maps at V_b = -0.90, -0.80, +0.12, +0.57, +1.70, +2.08, +2.32, and +2.53 V (6 x 6 nm^2). Spectra ((dI/dV)/(I/V)) of a 4-molecule island are shown in Fig. 3c of the main text. Corresponding (x,y)-dependent (dI/dV)/(I/V) maps are shown in Fig. S2 for different bias voltages. Spectroscopic differences between type A and type B molecules are seen at negative biases, unlike for the 12-molecule island.

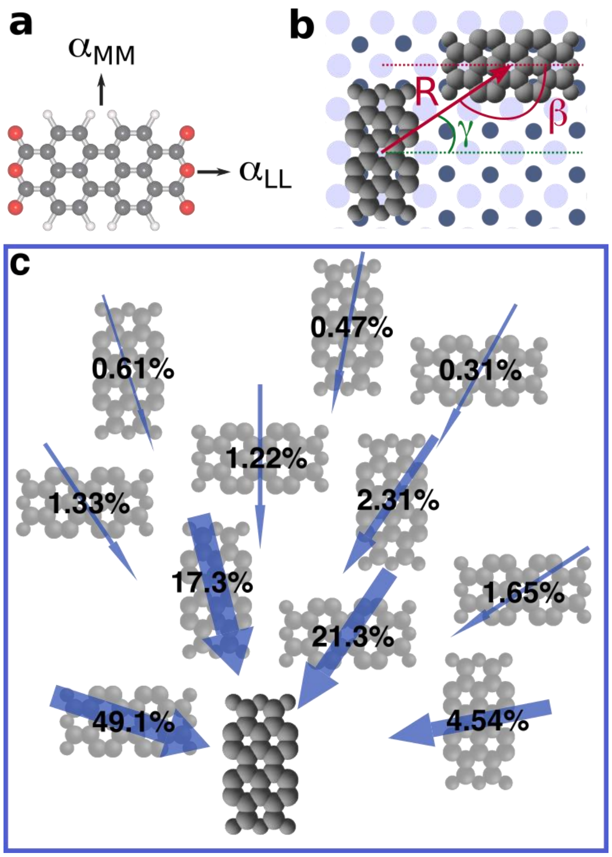

Supplementary Figure 3. Schematic of microelectorstatic polarizability calculations. a, In plane polarizability tensors of PTCDA molecule. **b,** point charge location determined by centre of PTCDA molecule on underlying NaCl lattice, showing angles used for microelectrostatic caluclations. **c,** Vector representation of induced dipole moment in the 12-molecule island with respect to molecule B1. Width of vectors corresponds to strength of induced dipole, percent contribution to Ep from each molecule is indicated.

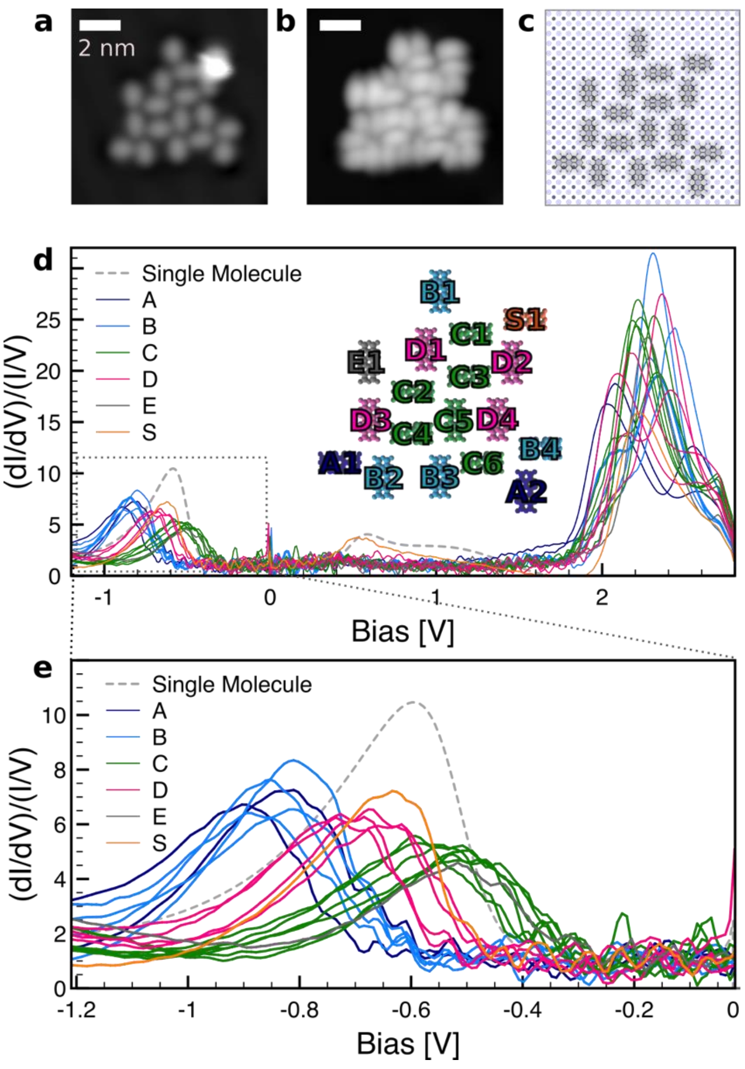

Supplementary Figure 4. (dI/dV)/(I/V) spectra of 18-molecule island on NaCl(2 ML)/Ag(111). a, b, STM constant current images of an 18 -molecule PTCDA island adsorbed on NaCl(2ML)/Ag(111), (9.5 x 9.5 nm^2, I = 30 pA, **(a)** V_b = +1.0 V and **(b)** V_b = -1.5V). **c,** Adsorption geometry of 18 molecule island showing location on NaCl lattice. **d,** (dI/dV)/(I/V) spectra of all molecules in the 18-molecule cluster with curves averaged over individual molecules identified in inset. **e,** STS of occupied states showing shifts of different molecular types, corresponding to the maxima used in Fig 5.

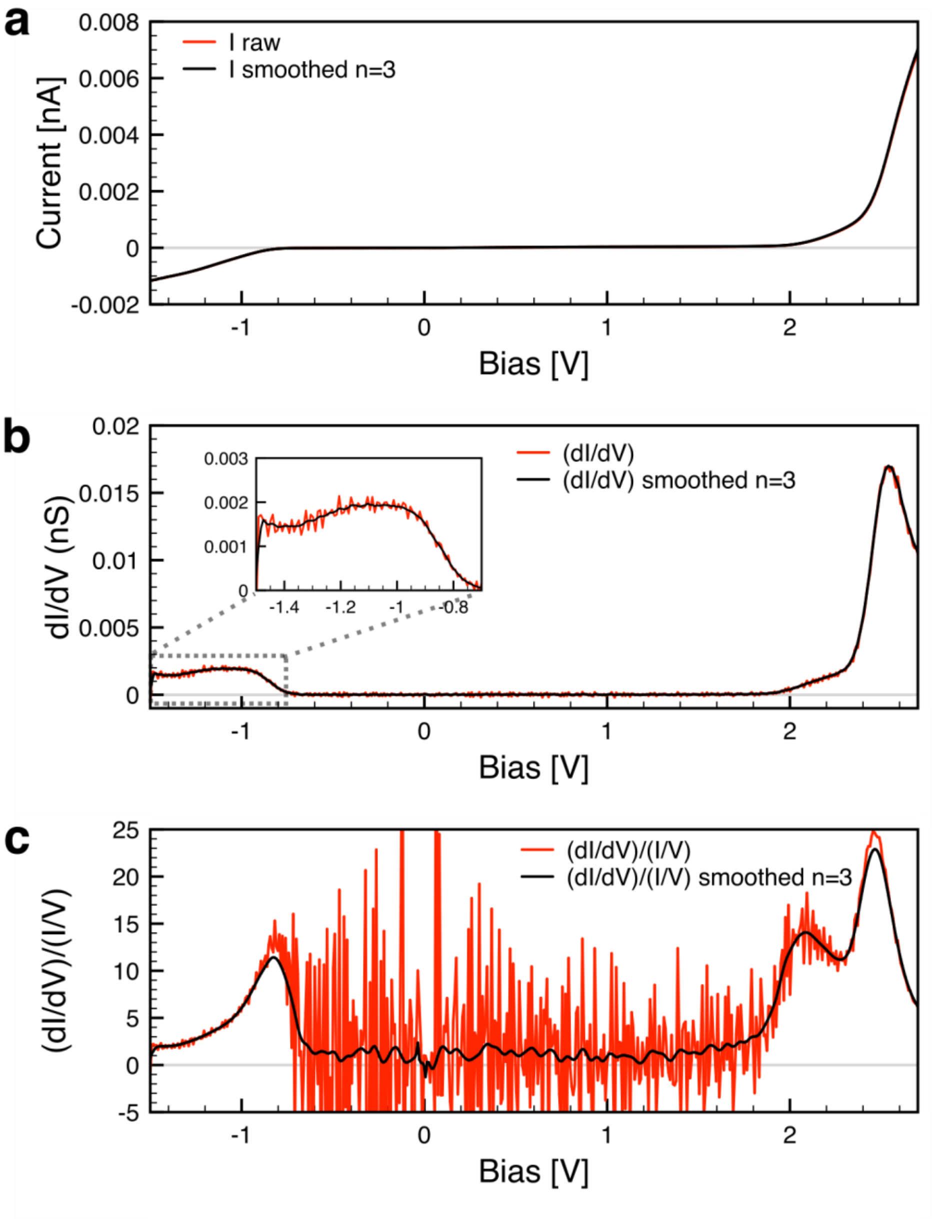

Supplementary Figure 5. Raw and N=3 moving averaged smoothed spectroscopy. a, I(V) **b,** dI/dV and **c,** (dI/dV)/(I/V) spectra of raw and smoothed data of a 12 molecule PTCDA nanoisland (A site) on NaCl(2ML)/Ag(111). We applied an n=3 boxcar moving average filter before computing the numerical dI/dV, yielding a bias resolution of $\Delta V*n/2=12$ meV. The smoothing procedure does not alter the location of the peaks or obscure any features, given the widths of the tunneling resonances observed. The smoothing aids mostly in the normalized spectra where the tunneling current is near zero.

Supplementary Notes

Supplementary Note 1: Adsorption position of PTCDA on NaCl (2 ML)/Ag(111)
The position of the PTCDA molecules on the NaCl(2ML)/Ag(111) lattice was based on the Cl^- top site position seen previously on bulk NaCl.[1] The lattice contraction of NaCl (2ML) on Ag(111) compared with bulk NaCl is minimal (Cl-Cl distance of 395 ± 6 pm and 390 ± 8 pm in two perpendicular directions, compared to the bulk Cl-Cl distance of 399 pm).[3] We found that all isolated PTCDA molecules adsorb at a 90° angle with respect to each other, and 45° with respect to the (100) salt island termination, consistent with the identical absorption to that found on bulk NaCl.

Supplementary Note 2: Negative charge of PTCDA on NaCl (2ML)/Ag(111)
Previously, Mohn, et al. have determined that PTCDA on NaCl(2ML)/Cu(111) is negatively charged.[7] This is explained by the small work function of NaCl (2 ML)/Cu(111)[8] in comparison with the electron affinity of PTCDA[9], which results in an electron transfer from the surface to the lowest unoccupied molecular orbital (LUMO). The work function of NaCl (2 ML)/silver is even smaller.[10,11] We therefore conclude that, in our case, the degree of population of the LUMO (and thus the amount of negative charge on the molecule) is the same as or larger than on NaCl (2 ML)/Cu(111). This negative charge state of the molecule is consistent with the repulsion and scattering of the NaCl (2 ML)/Ag(111) interface state electrons[3] by PTCDA (Fig S2e). Given the large Coloumb repulsion for a single molecule, relative to the LUMO-E_f difference, we expect the isolated PTCDA molecules to be in a 1^- charge state, rather than 2^-.

The formation of the clusters indicates that the H-bond network formed overcomes the Coulomb repulsion between the negatively charged molecules. The latter can be partially screened by the NaCl(2 ML)/Ag(111) interface state and Ag(111) conduction electrons, as has been observed for other polarizable surfaces in previous studies.[12,13]

Supplementary Note 3: Molecular position with respect to underlying Moire pattern
NaCl(100) bilayers on Ag(111) grow predominantly along the <112> direction. The 11% lattice mismatch between salt and silver lattices result in a Moiré pattern[4] [see (dI/dV)/(I/V) STS maps in Supplementary Fig. 1]. The latter corresponds to a spatial modulation of the work function, and therefore of the electrostatic potential. We determined the Moiré pattern at the location of the 12-molecule island (see Fig. 2 in main text) by using a Fourier filter[5] to remove the components corresponding to the PTCDA island from the (dI/dV)/(I/V)STS map in Supplementary Fig. 1a. That is, Supplementary Fig. 1b is a real-space image where the Fourier components related to the Moiré pattern in Supplementary Fig. 1a were exclusively conserved. The positions of the PTCDA molecules within the island with respect to this superstructure were deduced by superimposing the molecular model onto the STM topography and the Moiré interference lattice (see Supplementary Fig. 1d-f). We observe that electronically equivalent molecules (e.g., type A molecules in Supplementary Fig. 1e-f) lie on inequivalent sites of the Moiré [and therefore on inequivalent sites of Ag(111), although the adsorption sites of PTCDA on NaCl are all equivalent (see above)]. From this we conclude that the molecular spectroscopic properties are not influenced by the NaCl (2ML)/Ag(111) electronic structure. Defects surrounding the island (Supplementary Fig. 1a) are consistent with water clusters.[6] They also do not appear to perturb the intrinsic electronic properties of the molecules within the cluster.

Supplementary Note 4: 18 Molecule island

An 18-molecule PTCDA island was also examined, (Fig. 4,5 main text). Edge and centre molecules with the similar bonding configuration as in the 4- and 12-molecule islands were identified, as well as molecules with an intermediate level of coordination, which we label D. These molecules have an occupied resonance, O1 (Supplementary Fig. 3d, e), located in between that of the edge and the centre molecules. Two molecules do not show the expected behaviour. The molecule S has a spectrum similar to that of an isolated PTCDA molecule. In the positive bias imaging (Supplementary Fig. 3 a.) it appears as a bright spot, which appeared after a tip induced motion, and has a protrusion from the side, indicating it could be two PTCDA molecules adsorbed on top of each other. The other molecule, E, we attribute the unpredicted STS to lying on a defect of the underlying substrate, which also shifts the adsorption relative to the adjacent PTCDA.

Supplementary Method

Microelectrostatic Calculations

A simple microelectrostatic model was implemented in order to determine the energy shifts of electronic levels due to the local polarization of the neighbouring molecules for three islands. Each PTCDA molecule was treated as a point charge located at the centre of the molecule (Supplementary Fig. 4b), and the polarization of all other molecules was considered in computing the stabilization energy of that charge. The position and orientation of the molecules is known due to the known adsorption of PTCDA on the underlying NaCl lattice.

In general, the point charge representing the transient molecular ion creates an induced dipole moment, $\vec{\mu}$, in each of the other molecules:

$$\vec{\mu} = \alpha \cdot \vec{E} \tag{1}$$

with the electric field, $\vec{E}$, given by:

$$\vec{E} = \frac{e}{4\pi\epsilon_0 R^2}\hat{R} \tag{2}$$

where $\vec{R}$ is the displacement vector containing the charged molecule and the polarized molecule, *e*, is the charge of an electron, and ϵ_0 is the permittivity of free space. For convenience we write R for $|\vec{R}|$, and use $\hat{R}$ to represent the unit vector along $\vec{R}$, and use subscripts to denote the components of a vector in that direction.

To obtain the polarization stabilization energy, E_p,:

$$E_p = \left|-\vec{\mu} \cdot \vec{E}\right| \tag{3}$$

where by convention E_p, is positive, and reduces the ionization potential and increases the electron affinity. For a cluster, a sum of all contributing induced dipoles gives the total E_p.

For PTCDA, as with many organic molecules, the polarizability, α, is anisotropic and is represented by a tensor:

$$\alpha = \begin{pmatrix} \alpha_{MM} & 0 & 0 \\ 0 & \alpha_{LL} & 0 \\ 0 & 0 & \alpha_{NN} \end{pmatrix} \tag{4}$$

where *M*, *L*, and, *N* represent the short, long and normal molecular axes respectively. Here we only consider the two components α_{LL} and α_{MM} in the plane of the molecule as the molecules adsorb flat on the surface and there is no electric field component normal to the surface created by a point charge in-plane.

To compute the induced dipole, we must transform the electric field vector into the frame of the molecule, which yields the expression in the main text, Eq. 1. To instead work in Cartesian coordinates, we can write the expression for the induced dipole as:

$$\begin{pmatrix} \mu_x \\ \mu_y \end{pmatrix} = \begin{pmatrix} cos(-\theta) & -sin(-\theta) \\ sin(-\theta) & cos(-\theta) \end{pmatrix} \begin{pmatrix} \alpha_{MM} & 0 \\ 0 & \alpha_{LL} \end{pmatrix} \begin{pmatrix} cos(\theta) & -sin(\theta) \\ sin(\theta) & cos(\theta) \end{pmatrix} \begin{pmatrix} E_x \\ E_y \end{pmatrix} \tag{5}$$

where θ is the angle of the long axis of the molecule with respect to x, and E_x and E_y can be expressed as:

$$E_x = \left|\vec{E}\right| \cos\gamma = \left|\vec{E}\right| \frac{R_x}{R} \tag{6a}$$

$$E_y = \left|\vec{E}\right| \sin\gamma = \left|\vec{E}\right| \frac{R_y}{R} \tag{6b}$$

where γ is the angle of $\vec{R}$ with respect to the x-axis.

Due to the orthogonal orientation of the PTCDA molecules on NaCl(2ML)/Ag(111), with θ = 0 or 90°, we can simplify this to:

$$\mu_x = (A \cdot \alpha_{MM} + (1-A) \cdot \alpha_{LL}) E_x = \frac{e}{4\pi\epsilon_0 R^2}(A \cdot \alpha_{MM} + (1-A) \cdot \alpha_{LL}) \frac{R_x}{R} \tag{7a}$$

$$\mu_y = ((1-A) \cdot \alpha_{MM} + A \cdot \alpha_{LL}) E_y = \frac{e}{4\pi\epsilon_0 R^2}((1-A) \cdot \alpha_{MM} + A \cdot \alpha_{LL}) \frac{R_y}{R} \tag{7b}$$

with A=0 for a molecule aligned with the x-axis and A=1 for a molecule aligned with the y-axis. This results in a polarization energy due to the *ith* PTCDA molecule as:

$$E_{p,i} = \frac{e^2}{16\pi^2\epsilon_0^2 R_i^4} \left[(A_i \cdot \alpha_{MM} + (1-A_i) \cdot \alpha_{LL}) \left(\frac{R_{i,x}}{R_i}\right)^2 + ((1-A_i) \cdot \alpha_{MM} + A_i \cdot \alpha_{LL}) \left(\frac{R_{i,y}}{R_i}\right)^2 \right] \tag{8}$$

The contribution from each molecule is summed, and we can write:

$$E_{p,total} = \frac{e^2}{16\pi^2\epsilon_0^2} \left\{ \sum_{i=1}^{N_x} \left[(A_i \cdot \alpha_{MM} + (1-A_i) \cdot \alpha_{LL}) \left(\frac{R_{i,x}}{R_i}\right)^2 + ((1-A_i) \cdot \alpha_{MM} + A_i \cdot \alpha_{LL}) \left(\frac{R_{i,y}}{R_i}\right)^2 \right] \frac{1}{R_i^4} \right\} \tag{9}$$

which is equivalent to Equation1 in the main text, but only requires input of the x,y molecular coordinates and a coefficient to denote the orientation along x or y.

Supplementary References

1. Burke, S. *et al.* Strain Induced Dewetting of a Molecular System: Bimodal Growth of PTCDA on NaCl. *Phys. Rev. Lett.* **100,** 186104 (2008).
2. Guo, Q. *et al.* Adsorption of PTCDA on Terraces and at Steps Sites of the KCl(100) Surface. *J. Phys. Chem. C* **118,** 29911–29918 (2014).
3. Heidorn, S. *et al.* Influence of Substrate Surface-Induced Defects on the Interface State between NaCl(100) and Ag(111). *J. Phys. Chem. C* **117,** 16095–16103 (2013).
4. Heidorn, S.-C., Sabellek, A. & Morgenstern, K. Size Dependence of the Dispersion Relation for the Interface State between NaCl(100) and Ag(111). *Nano Lett.* **14,** 13–17 (2014).
5. Horcas, I. *et al.* WSXM: A software for scanning probe microscopy and a tool for nanotechnology. *Rev. Sci. Instrum.* **78,** 013705 (2007).
6. Guo, J. *et al.* Real-space imaging of interfacial water with submolecular resolution. *Nat. Mater.* **13,** 1–6 (2014).
7. Mohn, F. *et al.* Reversible Bond Formation in a Gold-Atom–Organic-Molecule Complex as a Molecular Switch. *Phys. Rev. Lett.* **105,** 266102 (2010).
8. Bennewitz, R. & Bammerlin, M. Aspects of dynamic force microscopy on NaCl/Cu (111): resolution, tip-sample interactions and cantilever oscillation characteristics. *Surf. Interface Anal.* (1999).
9. Chassé, T., Wu, C. I., Hill, I. G. & Kahn, A. Band alignment at organic-inorganic semiconductor interfaces: α-NPD and CuPc on InP(110). *J. Appl. Phys.* **85,** 6589 (1999).
10. Wang, H. *et al.* Band-Bending in Organic Semiconductors: the Role of Alkali-Halide Interlayers. *Adv. Mater.* **26,** 925–930 (2013).
11. Ploigt, H.-C., Brun, C., Pivetta, M., Patthey, F. & Schneider, W.-D. Local work function changes determined by field emission resonances: NaCl/Ag(100). *Phys. Rev. B* **76,** 195404 (2007).
12. Temirov, R., Soubatch, S., Luican, A. & Tautz, F. S. Free-electron-like dispersion in an organic monolayer film on a metal substrate. *Nature* **444,** 350–353 (2006).
13. Schiffrin, A. *et al.* Self-Assembly of l-Methionine on Cu(111): Steering Chiral Organization by Substrate Reactivity and Thermal Activation. *J. Phys. Chem. C* **113,** 12101–12108 (2009).
